# Professor Bartlett

**Published:** 1851-07

**Authors:** 


### PROFESSOR BARTLETT.

We were gratified to see it stated, soon after the issue of our last number, that Professor Bartlett had been appointed to the vacancy in the New York College of Physicians and Surgeons, occasioned by the death of Dr. John B. Beck. We were glad to learn it, both on his and the school's account. The institution could not have secured the services of a better man, and he has reached the place of his choice. Dr. Bartlett has for several years desired a residence in New York, and in the College of Physicians and Surgeons he is associated with some of his most genial and cherished friends. We wish him and his colleagues abundant success. Y.

